# What helps or hinders intervention success in primary care? Qualitative findings with older adults and primary care practitioners during a feasibility study to address malnutrition risk

**DOI:** 10.1186/s12875-024-02623-x

**Published:** 2024-10-23

**Authors:** Liz Payne, Elisabeth Grey, Michelle Sutcliffe, Sue Green, Caroline Childs, Sian Robinson, Bernard Gudgin, Pam Holloway, Jo Kelly, Jackie Seely, Rebekah Le Feuvre, Paul Aveyard, Paramjit Gill, Mike Stroud, Paul Little, Yardley Lucy, Leanne Morrison

**Affiliations:** 1 School of Psychology, University of Southampton, Southampton, UK; 2 Primary Care Population Sciences and Medical Education, University of Southampton, Southampton, UK; 3 The National Institute for Health and Care Research Applied Research Collaboration West (NIHR ARC West) at University Hospitals Bristol and Weston NHS Foundation Trust, Bristol, UK; 4 Population Health Sciences, Bristol Medical School, University of Bristol, Bristol, UK; 5 Dietetics Department, University Hospital Southampton NHS Foundation Trust, Southampton, UK; 6 Department of Nursing Science, Bournemouth University, Bournemouth, UK; 7 Human Development and Health, University of Southampton, Southampton, UK; 8 AGE Research Group, Translational and Clinical Research Institute, Newcastle University, Newcastle upon Tyne, UK; 9 Public and Patient Involvement, University of Southampton, Southampton, UK; 10 Nuffield Department of Primary Care Health Sciences, University of Oxford, Oxford, UK; 11 Warwick Medical School, University of Warwick, Warwick, UK; 12 Clinical Nutrition, University of Southampton, Southampton, UK; 13 School of Psychological Science, University of Bristol, Bristol, UK

**Keywords:** Person-based Approach, Malnutrition, Eating patterns, Ageing, Primary health care, General practice, Independent living, Health services for the aged, Dietary supplements, Feasibility study

## Abstract

**Background:**

In the UK, about 14% of community-dwelling adults aged 65 and over are estimated to be at risk of malnutrition. Screening older adults in primary care and treating those identified as ‘at risk’ may help reduce malnutrition risk and associated healthcare use, and improve quality of life. The aim of this study is to explore how primary care practitioners (PCPs) and older adults perceive, use and respond to an intervention to support those identified as ‘at risk’.

**Methods:**

We developed and optimised an intervention (screen and treat protocol, online tools and printed materials) to support primary care practitioners to identify malnutrition risk among older adults, and intervene where necessary. We recruited older adults (described as ‘patients’ here) taking part in a feasibility study, and carried out semi-structured interviews to assess PCPs’ and patients’ engagement with the intervention, and identify any contextual issues that supported or undermined their engagement.

**Results:**

Four themes were developed, encompassing patients’ and PCPs’ perceptions of undernutrition, study measures and appointments, constraints on PCPs’ enthusiasm to make a difference, and patients’ expectations of nutritional appointments. Key findings included patients commonly not accepting advice for undernutrition/malnutrition but welcoming support for their nutritional needs; checklists potentially distracting patients from recalling discussions about their nutritional needs; a tension between PCPs’ desire to recruit less-well patients and logistical difficulties in doing so; and patients compromising their nutritional needs to suit others.

**Conclusions:**

Diverse factors influence whether an intervention succeeds in primary care. PCPs learn about an intervention/study in different ways, vary in how they understand and accept its aims, and desire to make a difference to their patients. Patients bring perceptions and expectations about the study’s aims, coloured by their habits and preferences, prior experience of research and healthcare, and pressure from social expectations. Each aspect must be considered when developing a successful primary care intervention that is viewed as relevant and meaningful, and presented using language that aligns with participants’ values and goals. Our findings suggest that references to ‘malnutrition risk’ should be avoided in any patient-facing materials/interactions as participants do not accept or identify with this label.

**Supplementary Information:**

The online version contains supplementary material available at 10.1186/s12875-024-02623-x.

## Background

In the UK, about 14% of community-dwelling adults aged 65 and over are estimated to be at risk of malnutrition, rising to 18% of those receiving day care and home care [1]. Malnutrition is associated with increases in the likelihood of infection, falls, and depression [2], contributing to reduced quality of life, increased GP consultations and hospitalisation. The health and social care costs potentially attributable to malnutrition in England have been estimated as £19.6 billion (over the course of a year), which predominantly came from secondary healthcare provision to older adults [1]; this represented about 15% of the total health and social care expenditure.

Screening older adults in primary care and treating those identified as ‘at risk’ of malnutrition may reduce the resulting need for healthcare use [3], reduce infections and improve quality of life. Malnutrition risk is exacerbated by health and social conditions that are common among older people and which affect the sourcing of food, eating and/or absorption of nutrients. For example, social isolation and loneliness affect about 50% and 33%, respectively, of older adults [4] and have been found to increase the risk of malnutrition [5], likely due to their suppressing effect on appetite [6]. Primary Care Practitioners (GPs, nurses and healthcare assistants – PCPs) are trained to notice signs of weight loss among older patients with health conditions, but can lack training and confidence in how best to address appetite or weight loss [7, 8]. Support is therefore needed to help PCPs identify malnutrition risk among older patients and to provide appropriate treatment.

Older adults are aware that weight and appetite loss occur with deteriorating health and often frame this as inevitable age-related change [9]. The notion of risk from appetite or weight loss may also challenge older adults, for whom strong messages about risks associated with obesity, and personal responsibility for reducing weight, have been prevalent since the 1980s [10]. Interventions are therefore needed to support and address malnutrition risk in a way that is meaningful for older adults [11].

## Aims

We developed an intervention as part of the STREAM (Screen and TREAt for Malnutrition) project, incorporating training for PCPs and a package of ‘Eat well, feel well, stay well’ booklets for older adults at risk of malnutrition (Payne et al. [12]). The intervention aimed to encourage eating-related behaviour change by:

- Raising awareness of the connection between eating well and staying well;
- Providing tips and strategies for addressing commonly experienced barriers to eating and staying well, e.g. skipping meals, eating alone, beliefs about weight loss in ageing;
- Encouraging discussion around needs, priorities and goals;
- Offering suggestions and support to address identified issues.

We examined PCPs’ and older adults’ engagement with the intervention through a qualitative study nested within a feasibility study. The aim of the feasibility study was to assess whether participants could successfully complete study questionnaires and other study tasks, to check whether adjustments would need to be made before proceeding to a full trial.

In the present study, we aimed to identify experiences or contextual issues that undermined or supported PCPs’ and older adults’ engagement both in the study procedures and with the intervention by asking:

How do PCPs and older adults perceive, use and respond to the STREAM intervention?

## Methods

### Design

We used the Person-Based Approach to develop and optimise our intervention [12]. The Person-Based Approach is a systematic approach to applying qualitative research in intervention development [13], and seeks to understand individuals’ experiences and environment to address barriers and facilitators to engagement (see Band et al., [14] for details. The aim is to ensure that interventions are highly relevant and meaningful for those who will use them while retaining theory and evidence-based elements supporting beneficial behaviour change [15]. Hence interventions designed using the Person-Based Approach are more likely to be used, perceived to be useful, and effective.

The intervention was tested in a feasibility study, during which qualitative process evaluation interviews were carried out with PCPs delivering the intervention and patient participants (described as ‘patients’ from here onwards) receiving the intervention. Analysis of the patient data pertaining to the patient booklets, was published previously [12]. Data from the same patient interviews, but not previously published, is reported in the present publication. In particular, this includes patients’ reflections on paperwork, and in-person and phone consultations with nurses, related to the study. These reflections are considered in relation to PCPs’ reflections on the same components of the intervention, to assess how far these components meet patients and PCPs’ needs. The study is reported following COREQ criteria [16].

### Description of intervention

A prototype intervention was developed based on findings from our mixed methods synthesis [11], and exploratory interviews with older adults with malnutrition risk factors [9]. We knew from exploratory work that participants did not always identify with being ‘at risk’ [9]. Therefore, we named the intervention ‘Eat well, feel well, stay well’ and the content focused on strategies such as eating regularly and topping up food, to help ensure that users have energy to do everyday activities and stay well, rather than focusing on ‘risk’. Patient-facing information documents also avoided references to ‘malnutrition risk’. The prototype comprised a series of booklets, a food list and goal cards for patients. Details of the intervention development have been previously published [12].

A prototype online support tool for PCPs was developed, which included training to carry out feasibility study measures including grip strength; timed up and go test (TUGT); and nutritional assessment to assess current eating-related habits and issues, e.g. shopping, cooking and dental status (Additional file 2). The online tool also demonstrated how to screen patients using the Malnutrition Universal Screening Tool (MUST – a standard measure to assess the level of malnutrition risk [17], and deliver the intervention. Completion of the training was mandatory, after which PCPs could revisit the support pages as often as needed.

In the feasibility study, during an initial 20 min appointment, PCPs undertook the study measures, patients were screened using MUST, and some were also screened using the SNAQ (Simplified Nutritional Appetite Questionnaire) [18]. Those who had a MUST score of 1 or more and/or had a SNAQ score of 13 or less and/or had unintended weight loss in the last three months received our booklets along with up to four brief follow-up phone or in-person discussions over six months, depending on patient needs. Oral nutritional supplements were also provided, if needed.

### Participants

Eighteen face-to-face interviews were conducted with patients recruited via GP practice database searches, who were taking part in the feasibility study (ISRCTN76863664 Eat well, feel well, stay well… [19]. Patients were free-living adults aged 65 and over, meeting the following criteria which made them more vulnerable to risk of malnutrition:

- Chronic health conditions e.g. Chronic obstructive pulmonary disease (COPD), cerebrovascular disease; cardiac failure; chronic kidney disease (stage IIIb/IV/V); liver disorders; Parkinson’s disease; current depression, OR.
- Hospital stay in the previous 6 months, OR.
- Living alone.

All patient participants who were interviewed for this qualitative study had a MUST score or 1 or more, had a SNAQ score of 13 or less, or had unintended weight loss in the last three months.

Eight phone interviews were conducted with PCPs who carried out the study with patients in general practices.

### Procedure

Patients were identified via general practice database searches in South Central England. We anticipated needing to carry out around 10 PCP and 20 patient interviews, based on prior experience of intervention development. Those interested in participating completed a reply slip after receiving a participant information sheet and consent form. Researchers (LP, EG) phoned potential participants who were patients, and emailed PCPs, to confirm that candidates were happy to participate, and arranged interviews. Experienced qualitative researchers (LP, EG) carried out interviews, and three student interviewers (see Acknowledgements) were trained and supervised by experienced researchers (LP, LM). Consent forms were signed at the start of face-to-face interviews, or signed and returned by secure email before phone interviews. Spouses or carers could be present at patient interviews. Recruitment stopped once the research team agreed that adequate data had been collected to represent a range of views in the target population and participants were expressing no new addressable issues [20]. Patient interviews, lasting 22–66 min, took place between June 2018 and November 2018 and were conducted in patients’ homes one-three months after the PCP appointments. PCP phone interviews, lasting approximately 20–40 min, took place between September 2018 and May 2019. Interviewers probed how patients and PCPs experienced the appointments, screening, outcome measures and follow-up phone calls, based on interview topic guides (Additional files 1 and 2). Each interview was audio-recorded. Interviews were transcribed verbatim by a professional transcriber.

### Thematic analysis

Reflexive thematic analysis [21] of qualitative process evaluation data was carried out to explore how PCPs and patients perceived, used and responded to the study protocol and intervention. The aim was to assess how the intervention was delivered by PCPs and received by patients. Our critical realist perspective assumes that participants’ reported experiences reflect a real world, and that these are influenced by their psychosocial contexts. The analysis was conducted to enable rich interpretation of why particular aspects might support or undermine engagement, in the context of participants’ experiences of involvement in the study. We could then apply this interpretation in raising and appropriately addressing any issues identified before the intervention was evaluated in a large RCT.

Experienced qualitative researchers (LP, EG) familiarised themselves with the transcripts, then each transcript was coded in-vivo (i.e. codes derived directly from participants expressions about events, actions, values, beliefs or reflections) or from researchers’ understanding of participants’ expressions. Each transcript was coded by two researchers independently (LP: EG, LP: student) and differences in interpretation discussed. Experienced qualitative researcher (LM) joined discussions focused on interpreting and organising emerging themes in relation to the research question. Coding was likely also informed by researchers’ experience of previous data collected and analysed during early stages of the project [9, 11, 12], priming them to ‘notice’ issues (semantic meaning) and then consider the perceptions, experiences and contexts that may contribute to the construction of reported issues (latent meaning). The team’s varied experience of data collection and intervention development allowed a range of interpretations of the data, which could then be discussed. For the PCP data, a coding manual was developed to collate ideas and support the generation of themes, aided by MindManager version 20 [22]. For the patient data, a coding manual was developed, aided by NVIVO 12 (NVivo [23]). The coding manuals were amended throughout data analysis and reapplied to transcripts to ensure all aspects of participants experiences had been captured. Coded data excerpts were analysed by systematically retrieving and comparing excerpts relating to each code. These coded data were then grouped into themes, for example ‘Recognising and normalising undernutrition’ included data coded as ‘weight/appetite loss justified’ and ‘difference between support and advice’. Data from each theme are summarised narratively.

## Results

Participant characteristics are shown in Tables 1 and 2. Most patients were aged 65–84, half lived alone and few had recently been hospitalised. PCPs had a range of roles (Practice nurse, Research nurse, GP) and experience.

**Table 1:** Patient sample characteristics

**Characteristic**	***n*/18 (%)**
Age range (years) 65–74 75–84 85–94	8 (44) 9 (50) 1 (6)
Gender Female Male	11(61) 7 (39)
**Health conditions (self-report)** Cancer (not in current treatment) Cardiovascular Depression Gastrointestinal Musculoskeletal Respiratory Urinary tract	2 8 2 3 2 6 1
**Living alone**	8 (44)
**Recent hospital admission** **(last 6 months)**	2 (12)
Indicators of low appetite / malnutrition risk* MUST score = 1 or more Self-report MUST = 1 or more Nurse measured MUST = 1 or more SNAQ score = 13 or less BMI = 20 or less Unintended weight loss in last 3 months	10 (56) 6 (33) 6 (33) 12 (67) 6 (33) 9 (50)

*Self-report or nurse-measured *MUST* (Malnutrition Universal Screening Tool) and *SNAQ* (Simplified Nutritional Appetite Questionnaire), *BMI* (weight/height^2^).

**Table 2:** Sample characteristics of primary care practitioners (PCPs)

Characteristic	*n*/8 (%)
Gender - Female	8 (100)
Professional status Practice nurse Research nurse General practitioner	5 (63) 2 (25) 1 (13)

### Themes

**Fig. 1:**
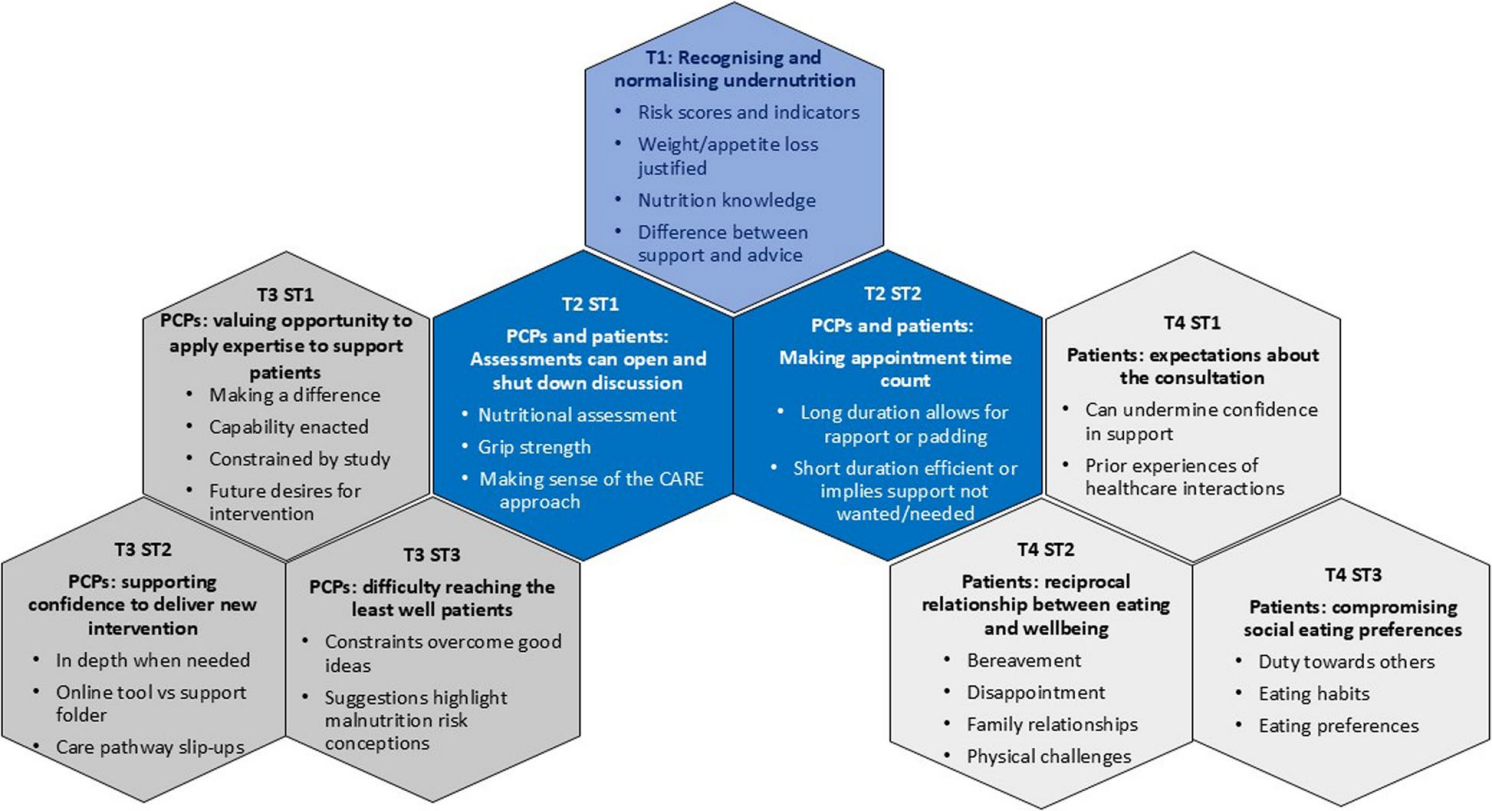
Theme summary

Four themes were developed from the data (Fig. 1). Theme 1 encapsulates alignment in patients’ and PCPs’ perceptions of undernutrition. Theme 2 identified a tension between PCPs’ and patients’ perceptions of the value of study measures and appointments – as either enhancing the conversation around undernutrition, or distracting from patients’ understanding and needs. Two further themes capture very different values and expectations that PCPs and patients bring to intervention appointments. Theme 3 includes three sub-themes demonstrating PCPs’ desire to make a difference to patients, while experiencing constraints on doing so; how they navigate the study actions to achieve this goal; and their creative ideas for recruiting the least well patients, which reveal more about perceptions of malnutrition risk. Theme 4 includes three sub-themes summarising patients’ experiences: expectations based on previous interactions with PCPs and healthcare systems; their experiences of food in relation to wellbeing; and the notion that their eating habits and preferences are already coloured by social expectations and compromise.

T1 = Theme 1, T2 = Theme 2, T3 = Theme 3, T4 = Theme 4, ST = Sub-theme.

Quotes below are from patients (PTxxx), PCPs (PCPxx), and Interviewers (I).

#### Theme 1: recognising and normalising undernutrition

Most patients did not identify appetite/weight loss or low BMI as reasons for participation, despite all participants having one or more malnutrition risk indicators (Table 1). A few thought that the study was aimed at people older than themselves, or those who lived alone or could not cook. Several stated that they were content with their current eating patterns and some expressed the view that the study and associated screening measures were not relevant to them.

*I found it really interesting….all these questions because they so don’t apply to me….to me they were such extraordinary questions to ask anybody.[PT072]*.

Instead, these patients wanted to help others, contribute to new knowledge through research, help the NHS, which had supported them through heart disease or stroke, ‘*do something different’* [PT067] to reduce depression about approaching the end of life, or simply oblige with the invitation to participate.

Both patients and PCPs seemed to consider the screen and treat intervention nonessential, with PCPs stating that most of the patients they screened were ‘fine’ based on MUST score. Some expressed disappointment about seeing patients who met the inclusion criteria but appeared not to need the intervention, *“Nice easy work for me*,* but umm….”[PCP03]*. One PCP commented that many patients’ main concern was being overweight, and for one patient, the concept of undernutrition was not considered a priority given the easy availability of food.

*The supermarket shelves are all full to overflowing….you’ve got to be financially capable of buying all this stuff*,* but….for me*,* it’s [undernutrition’s] not an important matter at all.[PT034]*.

Both patients and PCPs described reasons to explain weight or appetite loss, particularly physical activity, bereavement, infections, or health conditions that affect eating, e.g. irritable bowel syndrome. PCPs and patients were mutually unconcerned by risk of malnutrition identified by MUST scores, when a reason for low weight was identified:

*I had one patient that I saw for a second appointment*,* because she had a MUST score of 1….she wasn’t unhealthy*,* she wasn’t malnourished or anything*,* she was just very keen to stay fit and…eat healthily*,* because she’d had a TIA [transient ischaemic attack].[PCP05]*.

PCPs often attributed any subsequent improvement in appetite/weight to factors external to the intervention, such as that patients had been unwell and were now better. Equally, some patients seemed to resent assigning a ‘medical’ label to weight or appetite loss that they considered to be expected or psychosocial. For example, prior to participating in this study, two patients had experienced being told that they, or others around them in a hospital ward, had anorexia, and found this term aversive and unwarranted.

*I have been accused of being an anorexic and I’m not. Because I’m not ill*,* I’m not sick*,* so it’s just psychological somewhere on the line with me*,* which probably goes back a long*,* long way.[PT040]*.

Some patients did express concern or uncertainty about their weight and appetite loss or eating patterns, for example if they had made unsuccessful efforts to put weight on, or if their appetite or weight loss was recent and unexpected. Others were uncertain whether appetite loss was normal and what would constitute healthy nutrition.

*I could go all day without food but I get hungry at night. Is that pretty normal or.?….I’ll eat an apple each day. I tend to think….they’re good for you but I don’t really know.[PT150]*.

Several patients considered themselves knowledgeable about nutrition but PCPs perceived patients’ knowledge about nutrition and its impact on health to be inadequate, for example regarding the benefits of nutrition for wound healing. PCPs were therefore keen to impart their knowledge about this. However, patients expressed that they did not want eating advice or actions that they perceived as controlling. This was illustrated by their dislike of family members telling them to eat more despite foods being unappealing, manufacturers adding excessive sugar and salt to food, or Government advice which threatened the loss of specific pleasures.

*I: And how do you feel when your relatives are telling you what to eat? P: I do lose my temper a bit….Well*,* more than a bit….you do what you gotta do. Yes*,* I’ve had two heart attacks. Yes*,* I’ve got a stroke. But I’m seventy this year*,* I’ve got a good life. So*,* just leave me alone and let me eat*,* let me do what I want to do*,* you know.[p177]*.

Patients also indicated that they did not want advice they could not follow or to be told that they were eating the wrong things. Prior to this study, one patient had been told to avoid coffee, but had not found a replacement for it, so was drinking very little. Instead, patients responded positively to support, when there was a specific need for it, such as appropriate dietary changes to self-manage multiple health conditions, and PCPs demonstrating empathy for the individual’s circumstances and respecting the reasons for their eating difficulties. Some participants described the PCP as being thorough, or having a detailed discussion, which they perceived as *‘very good’[PT046]* and one assumed that the invitation was linked to their doctor noticing some weight loss at a heart follow-up appointment. These experiences with a focus on staying well appeared to help participants feel supported, perhaps enhancing their desire to engage with the intervention.

*My lifestyle [is] enhanced by feeling I mustn’t slide*,* because I’ve got this [intervention] backing me up now.[PT140]*.

However, one patient did express a preference for a more paternalistic style of intervention delivery.

*[The intervention] hasn’t really affected me that much. I hoped that it would*,* maybe someone would come along and tell me off because I hadn’t done something right.[PT074]*.

#### Theme 2: barriers and facilitators to meaningful discussions

##### Sub-theme 1: assessments can both open and shut down conversation

All PCPs used our nutritional assessment checklist (Additional file 3), which provided probing questions relating to eating difficulties, and signposted relevant supportive booklets. PCPs considered the nutritional assessment checklist useful for guiding discussion around eating, and for keeping patients focused on that topic: ‘*It generated conversation then about their eating habits’.[PCP04]* For example, one PCP described a patient who was pleased to discuss their loss of taste (an item on the form), which had not previously been addressed. PCPs also stated that the form helped them to explore potentially sensitive issues, such as finances, since patients had seen and completed the form prior to their appointment.

*There’s one [question] about benefits*,* claiming benefits*,* and I felt a bit uncomfortable asking that question*,* so*,* when they’d already done it that was quite good*,* so*,* I didn’t actually have to ask them.[PCP02]*.

In contrast, patients described the form as typical of checklists they had at hospital or general practice visits, suggesting that ticking the boxes may have been more memorable to them than any discussion of their nutritional needs that it may have triggered.

*Well she interviewed me*,* er and done a lot of ticking-off on the sheets and so on*,* after answering questions…. Umm*,* well they were general questions that any*,* well hospital*,* NHS or anyone*,* they would ask you*,* to try and find out who you were*,* and you know how things affected you and things like that.[PT034]*.

Both patients and PCPs expressed particular interest in a grip strength measurement, one of the study’s outcome measures carried out during the initial appointment, but not intended to contribute to the assessment or treatment of malnutrition. Grip strength measurement seemed to generate rapport and discussion; patients liked PCPs’ positive comments and encouragement about their scores, and PCPs reported that patients liked getting feedback. However, patients and PCPs appeared less clear on how these measures were relevant to nutrition.

*She said it was very good….despite the arthritis. So I was quite happy with that. I: Did you see the relevance of that? P: Well only that my hands work alright.[PT074]*.

*I didn’t know what a normal reading was or….[what] would flag-up a problem.[PCP04]*.

Not all PCPs adopted the CARE approach (Smith et al. [24]), promoted in the intervention package to facilitate supportive discussion to enable patients to make positive changes to their eating-related habits. Those who did, reported that the approach encouraged patients to ask questions, talk about intervention suggestions they had tried or challenges they had experienced. However, some PCPs stated that they were already experienced at talking with patients, and so did not feel the need to use the CARE approach.

*Congratulate*,* Ask*,* Reassure and Encourage [CARE]. Yeah*,* I think we probably used that loosely….I don’t think I referred to it at the time….I’m quite used to using a bit of motivational interviewing….where you try and ask more sort of open-ended questions and get people to come up with their own solutions of how they might tackle something.[PCP04]*.

##### Sub-theme 2: making appointment time count

PCPs commented that discussion was supported by the 20–30 min time allocation for study appointments, allowing them to develop rapport and discuss patients’ situations and needs. In contrast, a few patients mentioned that appointments were very short or that the PCP seemed busy, from which they concluded that there were no concerns about their eating and appetite, they did not need support, or that they had contributed little to the study. One patient commented that the initial appointment was too long and felt the nurse was trying to fill the allocated time. One PCP stated that it was important to address issues unrelated to the study as a way of respecting the patient’s participation.

*I just spent a little bit of extra time with [them]….I wanted to make sure that I had decent enough time slot*,* so that if there was a slight digression from what we were doing there was still plenty of time to get things done without feeling like you’re putting pressure on the person who….has volunteered their time to come in to do the research study.[PCP05]*.

Some patients appreciated the PCP contacting them for phone follow-up, to arrange follow-up appointments or discuss their individual eating needs, but one commented that the phone follow-up was an unnecessary burden on the NHS, and would have preferred to call the PCP at a pre-arranged time with any questions. PCPs also varied in how beneficial they found the phone follow-up, for example difficulties in contacting patients could undermine the PCP’s ability to offer support.

*One of them was a bit of a nightmare to get hold of. We agreed a time and date*,* and then when I started phoning we didn’t get any response….I did get hold of [them. They were] in a really noisy in the background*,* so*,* it was difficult to talk….I offered to phone back at a more convenient time*,* and apparently that was the more convenient time.[PCP01]*.

#### Theme 3: constraints on PCPs’ enthusiasm to make a difference

##### Sub-theme 1: valuing the opportunity to apply expertise to support patients

PCPs’ engagement with the study appeared to be driven by varied reasons. Several expressed enthusiasm, citing a belief that the intervention would inform appropriate care of those at risk of malnutrition, or enable them to teach or build relationships with patients. Some explained that they were trained to notice excessive weight loss, but previously lacked guidance on how to address this.

*Our trained nurses…are good at recognising umm weight loss and weight gain… unfortunately there isn’t always clear guidance in the surgery about what you should do afterwards.[PCP01]*.

Some used appointments as an opportunity to discuss nutrition more broadly, outside the study intervention guidance, e.g. giving advice about alcohol or explaining the ‘traffic light system’ used by food producers to show the relative fat, carbohydrate and protein content of foods. Some PCPs also offered the study’s booklets as a preventative tool to patients not identified as ‘at risk’, for example if they were lonely.

*There was….one [booklet] on feeling lonely*,* ‘Eating Alone’….I think it was from just chatting to the patient about their home situation*,* and perhaps picking up a problem that might affect their weight in the future*,* even though at the moment it doesn’t.[PCP04]*.

A few PCPs expressed their desire for implementation of study screen and treat activities in general practice, e.g. carrying out MUST assessments with all patients, using nutritional assessment checklists in clinics, and initiating discussions to pre-empt unintended weight loss. One PCP suggested incorporating MUST scores and nutrition discussions within a leg ulcer clinic.

*I was going to speak today at our nurses’ meeting about how we might free up a bit of time to have slightly longer dressing appointment and to maybe incorporate more nutritional screening into it.[PCP04]*.

##### Sub-theme 2: supporting confidence to deliver a new intervention

PCPs varied in how confident they felt in delivering the intervention, though most were positive about their ability to do this. Those with previous experience using the MUST, the CARE approach or discussing nutrition, stated that this made it easier, while those without such experience described feeling daunted. Individuals also described how study tasks suited their own characteristics, e.g. if they preferred to carry out study measures or discuss patients’ nutritional needs or both.

*It was quite nice*,* because there were practical things*,* so it wasn’t just sitting and chatting to people*,* so that was quite nice. ….it was quite an easyish sort of subject too*,* or I found it quite an easy subject to talk to patients about.[PCP11]*.

Gaining confidence in their ability to carry out the required tasks was attributed by some to having worked through the online support tool, while others favoured the study’s well-organised *“box of goodies”[PCP04]*, containing laminated key pages from the online support tool, patient booklets, grip strength equipment and brief reminders about actions to be taken during appointments. A couple of PCPs attributed their confidence to having read the patient booklets, while others stated that it became clearer once they put what they learned into practice.

*[With] the first patient I was very nervous; something new for me. But as the day goes on*,* I was feeling more comfortable and more confident.[PCP08]*.

Although PCPs were required to read all key pages in the online support tool before seeing patients, most described being selective about which pages they read and how thoroughly. One PCP only read the full details about the intervention when needed, e.g. once they had to give a patient oral nutritional supplements (ONS), and a couple asked colleagues to digest the content of the tool and explain the study to them. Those who liked the online support tool stated that they found it supportive, easy to navigate once it was familiar, and particularly useful for gaining new knowledge, e.g. how to measure grip strength. Those who found the online support tool challenging attributed this to the volume of content, particularly if much of it was new to them.

*It probably took me about an hour and a half*,* two hours in total….there’s quite a lot of reading in some of it as well and I like just to make sure that I have it all*,* you know*,* in my mind.[PCP05]*.

Different PCPs described using the online support tool and/or the support folder for revision before appointments, and during patient appointments, particularly when calculating BMI and MUST scores and following the required actions. PCPs’ views about the STREAM care pathway also varied - some stated that it was easy to follow, while one or two were confused about which patients should be given ONS.

*I: Have you dipped back into the online support tool at all? P: Very briefly. I’ve used the file [support folder] a lot to check things*,* especially if I had a query with a patient….I always took the file [support folder] with me….I’d take the whole box with me*,* so I can cover any eventuality with a patient to make sure I can give them the best possible….information and everything.[PCP05]*.

The patient booklets were well-received by most PCPs, as they were easy to understand and the ‘tick box’ sections helped expand discussions around patients’ home situations and reasons for eating well, and signposted other booklets to support patients’ specific difficulties. PCPs described the booklets as having supported their own understanding and confidence in delivering the intervention. However, not all PCPs were familiar with the booklet contents, while others seem to have selected booklets for patients, rather than involving them in a discussion as intended. Some patients seemed happy with this.

*I: I wondered if you had any feedback about those booklets at all? P: To be honest I don’t know that I even looked in them.[PCP11]*.

*I: And how did you choose the ones that you took*,* did you*,* or did [nurse’s name] just hand you? P: No*,* she just handed me the pack……were there others then? I: There are some others*,* but they’re for specific things like chewing and swallowing. P: Yeah*,* so that’s what she thought were most suitable for me. I: And what did you think about being given those? P: Good.[P092]*.

##### Sub-theme 3: Difficulty reaching the least well patients

PCPs suggested that study participation would be a low priority for patients who were frail, unwell or who relied on others to support their essential activities, and described barriers for less well-off patients, including transport costs or few friends and family to transport them. Fear that personal information would be shared outside the GP surgery, not liking paperwork, and not understanding what research involved were also identified barriers.

*A lot of people would have to come in via bus*,* and of course they would be reluctant to pay for their travel expenses….so*,* we’re not necessarily reaching out to the older population in the poorer sort of areas.[PCP05]*.

All PCPs had set up ‘screen and treat’ clinics on set days and times, as this was an efficient use of their time. The study protocol suggested screening and treating when patients attended other clinics, or during home visits, to reach frailer patients, but PCPs thought this would be logistically challenging. Some PCPs considered that the intervention should prioritise screening frailer housebound patients or those in care homes. One PCP suggested that patients who were able and willing to attend the surgery would be unlikely to need screening.

*What I feel is*,* this study must be more popular with probably nursing care homes….where we can find more malnourished patients.[PCP08]*.

Most PCPs seemed enthusiastic about opportunistic recruitment (identifying potential participants in routine GP appointments or nurse clinics), stating that this might identify more ‘at risk’ patients. Nurses suggested recruitment in clinics, e.g. leg ulcer, COPD and warfarin clinics, or among patients visited by proactive or elderly care teams, who would benefit from nutritional support. Some suggested other strategies, e.g. scanning appointment lists for suitable candidates. However, time constraints and the wellness of patients attending the surgery were described as key barriers to implementing opportunistic recruitment.

*Our GPs are*,* as are all GPs*,* quite busy*,* and we find it quite difficult to get them to refer patients to us and for them to remember it….We had sort of a bit of a busy patch as well*,* so*,* it was all a bit*,* you know*,* mad.[PCP02]*.

*We haven’t recruited anybody to STREAM that’s come from opportunistic recruitment ….because everybody that comes into the surgery doesn’t fit the criteria.[PCP03]*.

#### Theme 4: patients bring diverse expectations, degrees of wellbeing and social compromise to the intervention

##### Sub-theme 1: expectations about the consultation

Several patients described negative experiences during previous interactions with healthcare systems and professionals, unrelated to the current study, which may have reduced their confidence in receiving appropriate, supportive or useful discussions in the current study. For example, patients expressed irritation at being unable to reach their surgery to make appointments, and feeling hurried during appointments. Others sensed that PCPs could tend to be disinterested, e.g. after being advised to buy iron tablets at a pharmacy, or being *‘poo poo’d’[PT046]* when expressing views about pollution and COPD.

*And you get the feeling you’re boring the pants off them when you start to tell them anything*,* you’ve got a feeling they’re ooh*,* come on*,* you’ve got your seven minutes*,* hurry up and get out. So*,* it would be nice to be able to discuss it with somebody and not feel you’re wasting their valuable time.[PT166]*.

Negative psychological impacts of healthcare encounters were also described, which may have influenced expectations of treatment during the current study, particularly if previous health professionals were considered too blunt when giving a diagnosis or overly optimistic about possible outcomes of treatment, or used terminology, such as ‘heart failure’, which could be misinterpreted as the heart having stopped.

*He come straight out with it. One of them turned around*,* our first belief was you have probably got a brain tumour…. And when they couldn’t find anything wrong with me….they started to lose interest.[PT034]*.

Such experiences were described as negatively affecting eating. For example, one patient described being advised to urgently contact the hospital about growths found during an eye examination; the resulting distress and anxiety triggered a loss of appetite and seven kilos in weight. After investigations, no disease was identified, and the patient’s appetite returned. In contrast, one patient mentioned that hospital nurses ‘make it very easy’ to talk.

*I don’t mind talking to the nurse at all because they’re always very nice and they make it very easy.[PT095] (referring to frequent hospital appointments)*.

##### Sub-theme 2: reciprocal relationship between food and wellbeing

Some patients described learning to accept health problems, shed depression and be content with a more circumspect life as they got older. Experiences of pain, worry about themselves and others, and minor irritations such as being less able to open jars, were viewed as part of older age. Others expressed disappointment or sadness about having to adjust to their own or their spouse’s health challenges, including those who reduced their own activities to meet their spouse’s needs, suggesting that they might be open to ways to prevent decline.

*We went through a stage where I’d say ‘shall we have a drive out to [name of town]’….and she’d say ‘oh no why do you keep asking me?’ and I realised I’ve got to be the one to integrate….[PT094*,* interview with husband and wife PT094/PT095]*.

Disappointment was also expressed when health conditions made it impossible to eat previously enjoyed foods, perhaps enhancing the desire to discuss diet with a health professional, once the opportunity arose. For example, those on a gluten-free diet missed the taste of bread, another missed coffee and roast potatoes and found the food recommended for their health condition *‘uninteresting*,* bland and repetitious’[PT166]*. Some expressed an awareness that food choices could impact on wellness, having friends whose health was compromised, for example by not eating vegetables. One patient described regular meals with a relative who mocked the patient’s vegetarian diet, but then died from oesophageal cancer, which brought the patient great sadness and affected their eating.

*When he died*,* the week before last….I felt really unhappy and sad*,* and I didn’t want my dinner.[PT140]*.

Several patients described their happiness at having good relationships with children and grandchildren, but some expressed their sadness if family lived far away or visited infrequently. Others expressed their feelings of loss when a spouse, relative or friend died, leading them to spend more time alone at home or adversely affecting their sleep. Despite these expressions of sadness, most patients had not noticed a relationship between food choices and mood, though a few stated that being upset or worried would result in a reduced appetite, and one patient commented that specific foods seemed to affect their mood and energy. Others expressed their goal of keeping up their stamina, energy and appearance to ‘*make you feel alive’[PT140]*, or stated that they needed to be purposeful, for example by shopping for food, suggesting that they sought ways to support their wellbeing.

*I have noticed red meat gives me energy and improves my mood.[PT082]*.

*I: you said because of food shopping you can go out of the house. P: Yes*,* yes. It gives me a purpose to go out. That’s what I can get - a few things at a time.[PT065]*.

##### Sub-theme 3: compromising social eating preferences

Patients described very individual habits around eating with others, which might limit their ability to incorporate eating-related advice from PCPs, but also raised issues that could be discussed and addressed during a PCP consultation, supported by the intervention’s patient booklets. Patients described often *‘adapting’[PT040]* to others’ eating patterns to please them, perhaps reducing the opportunity to meet their own eating needs or preferences. For example, one patient described eating scones, because ‘*that’s what they[friends] like’[PT040]*, and another cooked for two brothers daily but did not eat with them, continuing their mother’s practice.

Frequently eating alone was not necessarily chosen but patients got used to it, and it then became a habit. It seemed to come about through circumstances, for example if patients were single when younger or divorced, or after a spouse’s illness, hospitalisation or death. Several patients commented that they preferred eating with others because they liked to socialise or disliked cooking. For example, one patient liked family meals at their daughter’s house, as this maintained a tradition and the daughter ensured that there was food that they could eat.

*I like doing that [family meals at daughter’s] but at home there’s almost nothing to like about it.[PT145]*.

Some also stated that they did not mind eating alone, describing making their *‘own little world’[PT040]* when eating, with television, newspapers or pets for company, or preferred to eat alone, for example after working alone for many years, or disliking talking while eating. When eating alone, many patients described being more likely to take their time, eat from a tray, snack, make easy-to-prepare meals, or choose something that only they would like, perhaps making it easier for them to adopt a PCP’s suggestions without taking others’ needs into consideration. Also, some desired to eat with others, but had not contemplated it once they got into a habit of eating alone, suggesting that they might be open to trying a new habit of eating with others.

*I: Would you like to eat more often with other people*,* with friends or? P: Yes*,* I wouldn’t mind. I’ve never really thought about it*,* because you get into that habit*,* you don’t….think it’s odd to eat on your own*,* do you.[PT049]*.

Several patients stated that they liked eating out, as restaurants offered a choice of food, and provided an enjoyable break from cooking and eating at home. Some described being encouraged to eat out by discounts from chain restaurants, while others ate out infrequently, for example at a supermarket café while shopping. However, some preferred *‘a good meal’[PT082]* at home, if they did not like restaurant menu options or mistrusted the pre-prepared food that they believed was available at restaurants. Others stated that restaurant visits were curtailed when health conditions made it too effortful, reduced menu options, or made their messy eating embarrassing. Nevertheless, some patients had strategies which enabled them to continue to eat out, such as a patient with coeliac disease who took their own gluten-free bread to restaurants, another who cooked and ate regularly with a friend who then reciprocated. A third enjoyed Sunday lunch at a *‘healthy pub’[PT140]* with others from their sheltered housing but also liked eating at home where they had more choice.

*I like eating at home because I*,* it’s the food I’ve cooked*,* and sort of favourite things*,* umm*,* but I do like have a meal out*,* of course I do*,* it’s nice and it’s social.[PT140]*.

## Discussion

The present qualitative study extends the literature by identifying experiences and contextual issues that undermine or support PCPs’ and older adults’ engagement with a malnutrition risk intervention in primary care. Four main themes were developed from the data. The first encompassed how nutrition knowledge and indicators of undernutrition were interpreted by PCPs and patients. It appeared difficult for both PCPs and patients to prioritise a change to eating patterns if patients were perceived as active, following a heart-healthy diet or enjoying social eating. This concurs with findings from our previous studies [9, 11] that older adults do not readily identify with the label of being ‘at risk’ of malnutrition. This also aligns with evidence that patients engage more effectively with interventions that focus on valued outcomes (e.g. maintaining fitness or independence) rather than challenging patients to accept ‘risk’ [25, 26]. In line with this, a key guiding principle in the design of the booklet-based component of the intervention was to emphasise how the nutritional advice will support wellbeing and independence, rather than challenging patients to accept malnutrition risk [12]. We also emphasised in our patient information that the advice was intended for people with (sometimes intermittent) low appetite, unintended weight loss, or lower than usual weight for their height and age-group, rather than for ‘malnutrition risk’ [12]. The risk-focused nature of the study measures and eligibility criteria challenged this guiding principle with both PCPs and patients commenting that measures of malnutrition risk (MUST score of 1 or more, SNAQ score of 13 or less, unintended weight or appetite loss) were not viewed as relevant or indicative of a problem that required action.

The second theme clarified how study measures, designed to quantify outcomes rather than contribute to the intervention, were experienced differently by PCPs and patients. Some PCPs stated that the nutritional assessment checklist, a study measure, enhanced their confidence by guiding the discussion about eating-related issues. A novel finding was that the checklist format of the form seemed to distract patients from eating-related discussion. Checklists are widely used in healthcare, to ensure that every relevant aspect is covered without PCPs needing to memorise each step [27, 28]. However, by focusing on medical issues that can be addressed, checklists can allow PCPs to avoid personal and affective issues, which they may find difficult or inappropriate to discuss but that may impact on the health condition [29]. Only one patient mentioned that the nutritional assessment checklist prompted a welcome question about their sense of taste, while most patients recalled the study measures, their impressions of the PCP or the duration of the appointment, rather than the content of discussion. This might imply that, for patients, open discussion focusing on their individual needs may be more important than answering checklist questions [28, 30]. Both patients and PCPs talked about the contribution of grip strength measurement to developing rapport and discussing needs, perhaps suggesting that its novelty, capacity to engender competitiveness or uncertainty about its purpose gave them common ground for conversation. It is also possible that the quantity of study and intervention components in the appointment may have overloaded patients [28]. Of course, intervention research also complicates the PCP’s role, as they must attend to study as well as intervention requirements, and different PCPs in our study preferred either carrying out study measurements or discussing eating, highlighting that combining the two can be cognitively challenging [31].

Our findings suggest that PCPs who used appointments to learn more about patients’ goals, home situation and specific eating-related priorities relating to health conditions, enabled patients to see value in a nutrition-focused intervention. This was particularly evident when patients differentiated between being given unwanted general advice and wanting support for issues that were important to them, agreeing with previous studies [32]. Some patients appeared to relish and benefit from participation in the study, for example gaining encouragement and suggestions to address their specific eating difficulties, which they seemed to have acted upon, suggesting that the nurse consultation worked well.

Themes three and four highlight how intervention components need to navigate the very different experiences and expectations that PCPs and patients bring to the nurse consultation. Theme three explores how PCPs valued flexible approaches to learning about and delivering the study. PCPs appeared motivated and enthusiastic to deliver the study, contrasting with previous studies which identified multiple barriers to malnutrition screening and treatment [11, 33]. To this end, PCPs differed in which intervention support tools they used, either the online support tool (use of which was mandatory), support folder, colleagues, or a combination of these. This extends previous literature by implying that, however well-structured the support tool, individuals need specific technical and language skills, learning styles and confidence to learn online [34]. PCPs reported that their confidence in understanding the study and delivering the intervention was enhanced by the study’s intervention tools, but some felt daunted until they had put their learning into practice with patients. This may have implications for fidelity to the study protocol, so encouragement to complete the online support tool was enhanced in the trial, along with adding extra crib sheets (e.g. for the CARE approach) to the support folder.

PCPs expressed concern about barriers to recruiting the patients with greatest need and explained that they would avoid inviting patients who would struggle to attend appointments, or become anxious and concerned that they might be malnourished. This may imply that PCPs’ professional obligation to protect their less-well patients can conflict with their role in implementing research [35], but can also override patients’ autonomy in choosing whether to participate [36] and increase health inequalities if those most in need are prevented from accessing support. Although PCPs suggested multiple ways to increase recruitment of less-well patients, who they considered may be more ‘at risk’ of malnutrition, they also described multiple barriers to doing this, including time-limits and the challenge of supporting those who could not attend appointments at the surgery. PCPs had not enacted any of their in-practice ideas, for example, identifying participants from certain clinics or from daily appointment lists.

Theme four reflects on how patient participants’ engagement with the intervention was shaped by their previous healthcare encounters and eating experiences. In this study, patients’ previous negative experiences, particularly with regard to communication with health professionals, seemed to engender a degree of mistrust, agreeing with previous research [37]. This could undermine patients’ confidence about receiving support or a reticence to fully engage with nutritional discussions.

Novel to this study, patients’ very individual eating-related preferences and habits often involved compromise while meeting the requirements of their health conditions, or others’ eating needs. Social norms are known to influence eating behaviours and the amount consumed [38], and it is not surprising that diverging from these norms can be challenging. Screening for malnutrition risk may add to the challenge, potentially disrupting not only individuals’ norms, but also their adaptations to their health conditions. Patients who take on others’ diets, sometimes to their own detriment, may be trying to model the new behaviour [39]or to ingratiate themselves [40]. Such examples evidence the need for the intervention to acknowledge patients’ varied needs and the associated challenge in using the advice.

Patients described continued enjoyment of social eating, yet some described the onset of difficulties which influenced the loss of social eating, which then became habitual. Previous studies emphasise the role of social eating in increasing food intake and enjoyment [41], and this topic seemed salient to participants. However, some patients enjoyed eating alone, agreeing with Thomas et al. [42], who describe those who prefer to eat alone as it gives them a sense of autonomy and peace. This suggests that it may be important to encourage changes to preferred habits, rather than assuming that everyone should be eating with others. This supports our decision to include in our booklets’ suggestions for both social eating and making eating alone enjoyable.

### Implications for research/practice

This study identified implications for future intervention design and testing in primary care. The aim was to intervene early with patients with malnutrition risk indicators, before these result in problems such as falls, infections and hospitalisation. However, PCPs seemed to downgrade the importance of lower levels of malnutrition risk identified with study measures (MUST), despite evidence that, in community settings, MUST is predictive of higher rates of hospital admissions and GP visits, and that appropriate nutritional intervention potentially improves outcomes. An RCT is currently being conducted to test whether the STREAM intervention, i.e. using SNAQ or low BMI or unintentional weight loss to identify participants, who are then assessed in primary care and given support if needed, results in improved outcomes (primary: quality of life, infections; secondary: timed up and go test; BMI; weight change etc.) over 12–18 months [43].

The ongoing RCT will contribute further evidence to support or refute the predictive value of MUST delivered in this population via GP surgeries. Also, future research must identify how soon on the trajectory of eating difficulties or weight or appetite loss PCPs should intervene, and which measures are most appropriate for identifying individuals who need support. For example, perhaps the onset of reduced social eating, or other elements of enjoyment, such as taste and swallowing could be early indicators of the need to intervene, before physiological detriments become evident and measurable.

Incorporating the required study outcome measures in the nurse-patient appointment may have distracted patients from engaging with the intervention, if they did not see it as relevant to themselves or if they felt insufficiently heard. In line with findings from this and our previous studies, that older adults do not identify with being labelled as having malnutrition risk, our approach in the intervention booklets was to focus on strategies such as eating regularly and topping up food, in order to have energy to do everyday activities and stay well, rather than focusing on ‘risk’, Future research should consider how the outcome measures can also be introduced in a way that aligns with participants goals and values, in this study identified as stamina, energy, appearance, purpose and enjoyment of life. PCPs in this study found that the nutritional assessment checklist helped guide the discussion, so it would be useful to find ways to support PCPs to cover all key elements and tasks while demonstrating to patients that they are not just receiving a standard set of healthcare questions that are replicated at every healthcare encounter.

PCPs in this study reflected on strategies to recruiting patients most in need of support who may not otherwise volunteer or be selected for invite, for example those who require a home visit. It may be fruitful to involve practice staff in developing the research question and recruitment plans early in the research. Further research investigating alternative approaches to inviting patients to participate in intervention studies would be useful, for example personalising invitations, and explaining the measures used.

In the present study, patients brought myriad different expectations to the PCP consultation, coloured by previous healthcare encounters, and emotional circumstances such as loss and bereavement. Patients had often changed their eating habits and behaviours to accommodate health conditions or others’ needs; the malnutrition risk intervention invoked further potential changes, which could add to the patient’s burden. Patients also described eating alone becoming a habit through circumstance, or preferred to eat alone. It is advisable that primary care interventions take into consideration the variability of patients’ history and avoid assuming that one size fits all, for example by asking about patients’ opinions, habits and preferences, and suggesting various options to support their specific needs.

### Strengths and limitations

The main strength of the study is that the experiences of both PCPs and patients with a range of ages, two genders and with different health conditions were included. A limitation is that data on ethnicity and deprivation were not collected.

The study relies on participants’ reports about appointments, capturing individuals’ personal experiences and reflections, both positive and negative. This allows the project team to better understand contextual issues, needs and expectations brought by PCPs and patients, which may impact on how well the intervention is accepted and implemented. A limitation of this is that memory can be imperfect, and participants may wish to please the interviewer or give what they perceive to be socially acceptable answers.

We have given a thorough description of the research methods used, in line with COREQ, to aid transparency.

## Conclusions

Diverse factors influence whether an intervention succeeds in primary care. PCPs learn about an intervention/study in different ways, vary in how they understand and accept its aims, but desire to make a difference to their patients, employing experience and professionalism. Patients bring perceptions and expectations about the study’s aims, coloured by their habits and preferences, prior experience of research and healthcare, and pressure from social expectations and compromise. Each aspect must be considered when developing a successful primary care intervention that is viewed as relevant and meaningful, and presented using language that aligns with participants’ values and goals. Our findings suggest that references to ‘malnutrition risk’ should be avoided in any patient-facing materials/interactions as participants do not accept or identify with this label.

## Supplementary Information

Additional file 1: Interview Topic Guide (Patients)

Additional file 2: Interview Topic Guide (PCPs)

Additional file 3: Nutritional assessment checklist

## Data Availability

The datasets supporting the conclusions of this article are included within the article and its additional files.

## Abbreviations

COREQ: Consolidated criteria for reporting qualitative research
MUST: Malnutrition Universal Screening Tool
RCT: Randomised Controlled Trial
SNAQ: Simplified Nutritional Appetite Questionnaire
