# Supplementary material for: What helps or hinders intervention success in primary care? Qualitative findings with older adults and primary care practitioners during a feasibility study to address malnutrition risk
Source: BMC Prim Care. 2024 Oct 23;25:377. doi: 10.1186/s12875-024-02623-x (PMC11515772; doi:10.1186/s12875-024-02623-x)
Supplement: Supplementary file 1 — Additional file 1: Interview Topic Guide (Patients) [file 12875_2024_2623_MOESM1_ESM.docx]

**Additional file 1: Interview Topic Guide (Patients)**

This interview guide is designed to ask about patients’ experience of taking part in the Eat well, feel well, stay well feasibility study. This will include asking patients about their appetite and eating patterns and habits, their views about booklets and other materials we have developed, and how it was taking part in the study. Patients’ views will help us to improve the materials, and how malnutrition screen and treat policies are delivered in doctors’ surgeries, which will then be tested in a randomised controlled trial.

The interview guide will evolve over time based on responses from early interviews, to ensure that questions are relevant to, and understood by participants.

| **General open questions** | **Possible probing / prompting questions**  (to use in response to participants’ comments) |
| --- | --- |
| 1. I’m interested to hear about your appetite – could you tell me how you are finding this at the moment? | - Could you describe what you like to eat and drink? - Could you tell me about the sort of things that affect your appetite? - Could you describe times and places when you tend to feel hungry or thirsty? - When you feel hungry or thirsty, can you talk me through what you do? |

| **Questions about being in the study** | **Probing questions** |
| --- | --- |
| 1. I’d be really interested in how you found the information you were sent about this study. Could you tell me about this? | - Can you talk me through the different information you had about the study? - Can you tell me what you thought of the information you were sent – both positive and negative? |
| 1. I’m very interested to find out what it was like being in the study. Can you tell me a bit about that? | - Could you tell me what made you want to take part? - Can you talk me through what you have done in the study? |
| 1. I’m interested to hear how you found it filling in the list of questions *like this one (show example questionnaire)* that you were sent. Could you tell me about this? | - What did you think of the questions? - What did you like about the question list? - What did you like less about it? - We are thinking of changing some of the questions – could you take a look at the new questions and let me know what you think of them? - How easy would you find it to answer the new questions? - How relevant are the new questions to you? - Which of these are most important for you? - What other things are important to you? E.g. improve appetite? Quality of nutrition in what you eat? Not getting frail? Getting less infections? Anything else? |
| 1. I’m really interested to hear about your appointment(s) with the nurse / doctor. Could you tell me about this? | - Can you tell me about any appointments you have had with the nurse / doctor as part of the study? - If you had a phone appointment with the nurse / doctor, can you tell me what that was like? - Can you talk me through what happened at the appointment(s)? - Did the nurse/doctor ask about your appetite and eating patterns? If so, how did you find this? - Will you have any more appointments? - Did you make the appointment with the nurse or did the nurse phone you? |
| 1. I think you were given a form to fill out about your general health like this one (show example*(show example nutritional assessment checklist – patients’ version)*. I’d be interested to hear how you found that*?* | - Can you tell me about anything else that might be useful to add to this? - Can you show me which parts are most relevant to you? |
| 1. I’m interested in whether you were given or tried special drinks from your doctor. Could you tell me a bit about this? | - If yes, can you describe how this came about? - If no, have you ever had special drinks? - Can you tell me how you find / found the special drinks? - I’m interested to know what your appetite is / was like while you are / were taking special drinks. Can you tell me about this? - I’m interested to know what your eating patterns are / were like while you are / were taking special drinks. Can you tell me about this? - What about drinks you can buy from the pharmacy, such as Complan? Have you ever tried these? Can you tell me about this? |
| 1. *Only for people who offered to give urine and / or blood samples as part of the study* | - If you gave some urine samples, can you talk me through how you found that? - If you gave some blood spot samples, can you talk me through how you found that? - If you didn’t, could you say why? |
| 1. Is there anything you’d like to change about your eating habits? |  |
| 1. Is there any advice about eating that you would find useful? |  |
| 1. Have you had an appointment with a different nurse to measure your grip strength? | - If so, how did you find it? |

**Thank you: is there anything else you would like to say?**
