# Supplementary material for: What helps or hinders intervention success in primary care? Qualitative findings with older adults and primary care practitioners during a feasibility study to address malnutrition risk
Source: BMC Prim Care. 2024 Oct 23;25:377. doi: 10.1186/s12875-024-02623-x (PMC11515772; doi:10.1186/s12875-024-02623-x)
Supplement: Supplementary file 2 — Additional file 2: Interview Topic Guide (PCPs) [file 12875_2024_2623_MOESM2_ESM.docx]

**Additional file 2: Interview Topic Guide (PCPs)**

This interview schedule is designed to ask about health professionals’ experiences of taking part in the ‘Eat well, feel well, stay well’ feasibility study. The feasibility study consists of General practice staff doing an online training session, then screening older adults to find those with low appetite or eating and drinking patterns that may affect their health, offering support, including printed materials, and following a care pathway. The aim of the online and printed materials is to encourage patients to eat and feel well, and to help health professionals to support patients in this.

**Further questions may be added to the schedule as the intervention is developed**. This will ensure that the questions are relevant and easily understood by participants. It will also allow us to include questions based on comments by participants in early interviews.

| **General open questions** | **Possible probing / prompting questions**  (to use in response to participants’ comments) |
| --- | --- |
| 1. Can you tell me about your experience of supporting patients in the study? | - Can you tell me, overall, how you found it supporting the patients? - Can you describe how you decided what support to offer for different patients? - Were there any things that made you decide not to carry out screen and treat with some patients? |
| 1. I’m really interested in how you are finding using the materials / booklet for patients. Can you tell me about this? | - Can you tell me, overall, how you found the materials / booklet(s)? - Can you talk me through how used the materials for patients? - Can you tell me how you found it using the materials / booklet(s) with patients? - Can you describe how patients responded when you used the materials / booklets with them? - Can you describe how carers/family members or others responded if you used the materials / booklets with them? |
| 1. I’m also interested in what you thought about the materials / booklets for patients. | - Can you tell me about anything you thought was good about the materials / booklet(s)? - Can you tell me anything about the materials / booklet(s) that you were less keen on? - Could you tell me about anything you think might make it easier for patients / people who help them out to follow the suggestions shown in the materials / booklet(s)? |
| 1. I’m interested to hear how it been following the guidance given in the materials for staff. Can you tell me a bit about this? | - Can you talk me through how you used the online / printed materials for staff? - Can you tell me about anything you thought was good about the online / printed materials? - Can you tell me anything about the online / printed materials that you were less keen on? - Can you show me which parts were most relevant to you? - Can you suggest any ways that these online / printed materials might be improved? |
| 1. How has it been for staff at your practice to follow the processes and procedures in ‘Eat well, feel well, stay well’? | - Can you describe how your practice found it using the suggested processes and procedures? - Can you tell me about any parts of the processes and procedures that your practice found useful? - I’m interested in hearing about any parts of the procedures that your practice changed? Can you tell me about this? - Could you tell me about anything that would make it easier for your practice to follow the procedures in the intervention? |
| 1. Can you tell me how has it been supporting patients face to face / by email / by phone / the combination of these? | - Could you talk me through a typical face to face appointment? - Could you talk me through a typical phone follow-up? - Can you describe what you have you liked about supporting patients using these methods? - Can you describe what you have you been less keen on when supporting patients using these methods? |
| 1. How are you finding supporting patients using the new materials compared to your previous processes? | - Can you tell me about any ways that the new materials have helped? - Can you describe any ways that the new materials have been less helpful? - Can you suggest any improvements to the new materials? |
| 1. I’m interested to know how you found it giving oral nutritional supplements (ONS), (if you did). Could you tell me about this? | - Could you talk me through a typical discussion about ONS? - Can you tell me how you found it talking to patients about using ONS? - How did patients respond when you talked to them about using ONS? - Can you suggest any ways that would make it easier to explain about using ONS? |
| 1. I’m interested to hear how you found it using the CARE approach with patients. Could you tell me a bit about that? | - Can you tell me about ways you used the CARE approach? - Can you tell me how patients responded to the CARE approach? - Can you say what you liked about the CARE approach? - Are there any things you were less keen on about the CARE approach? - Can you suggest any ways that would help nurses to use the CARE approach? |
| 1. Can you tell me about anything else that might be useful for you? | - I’m interested to know of any ways you think the service might be better delivered? - Can you suggest anything else that might help to deliver the intervention (eg. Tools, guidance)? |
