# Supplementary material for: What helps or hinders intervention success in primary care? Qualitative findings with older adults and primary care practitioners during a feasibility study to address malnutrition risk
Source: BMC Prim Care. 2024 Oct 23;25:377. doi: 10.1186/s12875-024-02623-x (PMC11515772; doi:10.1186/s12875-024-02623-x)
Supplement: Supplementary file 3 — Additional file 3: Nutritional assessment checklist [file 12875_2024_2623_MOESM3_ESM.docx]

**Additional file 3: Nutritional assessment checklist**

These are possible actions you can take as a result of a patient’s answers to these questions

| To save time, ask patients to complete Sections 1-5 of the checklist before their appointment. If they have not done this, you may like to ask them to complete it while in the waiting room. | | |
| --- | --- | --- |
| **Topic** | **Answer** | **HCP notes** |
| *Section 1: Eating and drinking* | | |
| How many meals do you have per day? | 🞎 None  🞎 1 meal  🞎 2 meals  🞎 3 meals  🞎 4 or more meals |  |
| How many snacks do you have each day? | 🞎 None  🞎 1 snack  🞎 2 snacks  🞎 3 snacks  🞎 4 or more snacks |  |
| How many cups or glasses do you normally drink in a day? Please include any liquid (tea, coffee, water, juice…) | 🞎 None  🞎 1-2 cups or glasses  🞎 3-4 cups or glasses  🞎 5-6 cups or glasses  🞎 7-8 cups or glasses  🞎 9 or more cups or glasses |  |
| Has your appetite changed recently/in the last few weeks? | 🞎 Yes  🞎 No If yes, please explain how: | Offer Booklet 1 |
| Do you think any of your medications make you eat or drink less? | 🞎 Yes  🞎 No If yes, please explain how: | Offer Booklet 1  Check if medication review is needed |
| *Section 2: Eating experience* | | |
| How do you find it when chewing? | 🞎 I can chew all food well  🞎 I find chewing soft food easier | Refer to dentist or speech therapist and offer Booklet 2 |
| How do you find it when swallowing? | 🞎 I can swallow food  🞎 I can swallow liquids | Refer to dentist or speech therapist and offer Booklet 2 |
| *Section 3: Mouth care* | | |
| Do you have dentures? If yes, how do you find them? | 🞎 I have dentures  🞎 My dentures fit well  🞎 My dentures do not fit well | Refer to dentist or speech therapist and offer Booklet 2 |
| Do you have any problems with your teeth and mouth? | 🞎 Yes  🞎 No If yes, please explain how: | Refer to dentist or speech therapist and offer Booklet 2 |
| *Section 4: Shopping and cooking* | | |
| Does anyone help you to do you shopping or prepare your food? | 🞎 I do my own shopping  🞎 I get help with shopping  🞎 I get my shopping delivered | Refer to local support services and offer  Booklet 3 |
| Where do you mainly get your shopping? |  | Refer to local support services and offer  Booklet 3 |
| Does anyone help you cook your food? | 🞎 I do my own cooking  🞎 My spouse/carer does the cooking  🞎 I get cooked food delivered  🞎 I have cooked food out (e.g. Luncheon club)  🞎 I don’t have cooked food | Refer to local support services and offer  Booklet 3 |
| If there is someone who helps you with daily activities (such as cooking or shopping), you can bring them along to the first appointment. | |  |
| *Section 5: Daily living* | | |
| Do you have any government allowances? | 🞎 Attendance allowance  🞎 Disability living allowance  🞎 Other: | Refer to local support services |
| Do you ever eat less than you want or skip meals because of the cost? | 🞎 Yes  🞎 No | Refer to local support services |
| Do you have any concerns about managing your money? | 🞎 Yes  🞎 No | Refer to local support services |
| Do you live by yourself? | 🞎 Yes  🞎 No | Refer to local support services and offer  Booklet 4 |

| To be filled out together with patient.  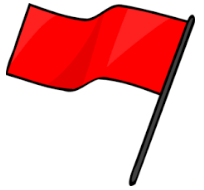Some symptoms may warrant particular clinical attention and  referral, if the answer is yes to questions marked with a red flag. | | | |
| --- | --- | --- | --- |
| Topic | Answer | HCP notes |  |
| *Bowel habits* | | | |
| Have you had any change in bowel habit in the last 3-6 months? | 🞎 Yes  🞎 No If yes, please explain how: | 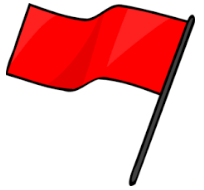Refer patient to other health professional if clinical need |  |
| Have you had any blood in your stools? | 🞎 Yes  🞎 No | 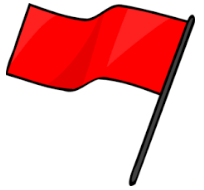Refer patient to other health professional if clinical need |  |
| Do you sometimes feel you have not emptied your bowel? | 🞎 Yes  🞎 No | 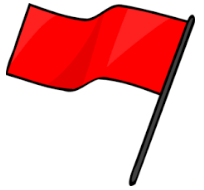Refer patient to other health professional if clinical need |  |
| Do you have any pain in your stomach? | 🞎 Yes  🞎 No | 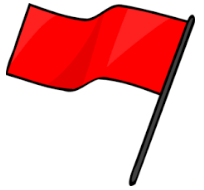Refer patient to other health professional if clinical need |  |
| Do you experience any nausea? | 🞎 Yes  🞎 No | 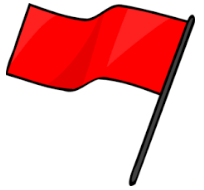Refer patient to other health professional if clinical need |  |
| *General health* |  |  |  |
| Do you feel pain? | 🞎 Yes  🞎 No If yes, please explain where: | Refer patient to other health professional if clinical need  Check prescription and see if **medication review** is needed |  |
| Do you have a cough? | 🞎 Yes  🞎 No  If yes, how long have you had the cough?  🞎 1 week  🞎 2 weeks  🞎 3 weeks  🞎 More than 3 weeks | Refer patient to other health professional if clinical need |  |
| Do you ever cough up blood? | 🞎 Yes  🞎 No | 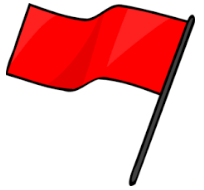Refer patient to other health professional if clinical need |  |
| Have you noticed any lumps or swellings under your skin? | 🞎 Yes  🞎 No | 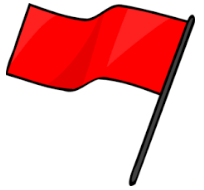Refer patient to other health professional if clinical need |  |
| Do you feel tired? | 🞎 Yes  🞎 No | Refer patient to other health professional if clinical need |  |
| Do you feel depressed? | 🞎 Yes  🞎 No  If yes, how long have you felt depressed?  🞎 1 week  🞎 2 weeks  🞎 3 weeks  🞎 More than 3 weeks | Refer patient to other health professional if clinical need |  |
